# Human resource technology disruptions and their implications for human resources management in healthcare organizations

**DOI:** 10.1186/s12913-019-4068-3

**Published:** 2019-04-29

**Authors:** Aizhan Tursunbayeva

**Affiliations:** eHealth Research Group, Usher Institute of Population Health Sciences and Informatics, The University of Edinburgh, Teviot Place, Edinburgh, EH8 9AG UK

**Keywords:** eHealth, Human resources, Health care management, Information systems, Human resource information systems, HRIS, People analytics

## Abstract

Concern among the public and policymakers about current and future major staff shortages is increasing. Strengthening Human Resource (HR) practices and adopting HR technologies such as Human Resource Information Systems (HRIS), that can collect, store and report workforce data are often described as a potential solution to this problem. Indeed, examples from other industries show that HRIS can help to launch or manage, as well as provide ongoing insights concerning the whole career cycle of an employee. However, few of the existing studies that discuss technology or its impacts on the future of work have focused on health organizations, and those that do have not received sufficient attention in health literature. Furthermore, such contributions as there have been have either prioritized a particular type of technology or focused mainly on the effect of automation on health professionals’ work. They have thus overlooked the full range of possible uses of these technologies and, specifically, have neglected the topic of HR for Health (HRH) management in health organizations. The primary aim of this paper is to address this lacuna, with specific reference to the existing categorization of HR technological disruptions. To conclude, health organizations and the health and HR professionals who work within them need to use HRIS responsibly, finding a balance between the drive for innovation, productivity and efficiency and respect for all potential legal, ethical and compliance issues, as well as taking account of the importance of HRH wellbeing and satisfaction.

## Background

Concern among the public and policymakers regarding current [1] and future major HRH shortages, predicted to reach 12,9 million professionals worldwide by 2035 [2], is increasing. These worries are often associated with awareness of a growing demand for chronic care, on the one hand and a recent tendency toward burnout among health professionals [3]. Appropriate management, development, and retention of health professionals are frequently mentioned as critical factors both in addressing this challenge, and also in facilitating effective management of health organizations, and improvement in the quality of patient care. Nevertheless, many health organizations persist in archaic HR practices and the role of HR technologies is conceived as being to support existing ways of doing things. Some of them even struggle to estimate timely and accurately the size of their HRH [4]. HRIS [5], proven in other industries to be effective in transforming selection, management, development and retention of employees, are often thought of as a potential panacea for weaknesses in HR systems and practices in health organizations. It is expected that HRIS can help to launch, manage, and provide actionable insights on a full employee life-cycle, starting from recruitment and selection, and continuing through payroll and benefits administration, performance management, career and succession planning [6]. However, little of this work has focused on health organizations or published in health literature. In consequence, knowledge regarding the adoption and impacts of HR-related innovative technologies in health organizations is still scarce.

Moreover, the rapid developments in the field of technology over the last decades referred to by the World Economic Forum as the “fourth industrial revolution” - after steam, mass production, and electronics – have given rise to a wide variety of technologies and their possible applications in HR management or development. However, those few studies that discuss HR technologies in healthcare focus only on limited types of technologies such as, for example, Artificial Intelligence (AI) (e.g. [3]). This paper contends that a wide range of HR technologies can, in principle, support and improve HR practices in healthcare and thus help to address the upcoming HRH crisis. In reviewing this the paper draws on the existing typology of innovative HR technology disruptions that could transform the way we work and manage organizations [7] including: 1. The acceleration of HR Management Systems (i.e. HRIS) and cloud-based solutions; 2. A massive shift from “automation” to “productivity”; 3. The introduction of continuous performance management processes; 4. The rise of feedback, engagement, and analytics tools; 5. A reinvention of corporate learning; 6. Innovation in the recruitment market; 7. An explosion in the wellbeing market; 8. The maturation and growth of the “People Analytics” market; 9. The spread of intelligent self-service tools; and 10. Innovation within HR itself.

The examples used in this paper come mostly from the background reading of the author undertaken in the course of research on HRIS in health and “People Analytics”.

### HR technologies that could transform HRH work and management

#### The acceleration of HRIS and cloud-based solutions

Health organizations first adopted HRIS to automate manual payroll activities back in the 1960s [8]. Today most health organizations use HRIS to support operational core HR practices such as HR administration, time/absence monitoring, rostering/scheduling, employee benefits or expenses management. However, there is evidence that some health organizations also use HRIS to enable more strategic HR practices such as employee recruitment, performance, learning and development management, as well as succession or workforce planning [9]. At the same time, other innovative HRIS have begun to emerge [7]. This is a result not only of cutting-edge technological developments, and of the emergence of new vendors, but also of a move by some technology giants into the HR technology space.

The on-premise nature of the HRIS that dominated the HR systems market for many years is also changing as subscription-based cloud have become available [7]. Sierra-Cedar [10] has projected the number of health organizations using the cloud to grow from 29 to 51%, while the number of licensed deployments was supposed to decrease from 75 to 62% by 2015. The two primary reasons for this shift were easier upgrades (31%) and improved user experience (25%). Tower Watson’s perspective [11] also concluded that 60% of large health providers announced operational improvements to their centrally managed employee service centers by making them cloud-based.

#### A massive shift from “automation” to “productivity”

One of the initial aims of HRIS was to automate paper-based processes. However, there is now an emphasis not just on automating certain tasks, but on increasing productivity generally by helping individuals and teams to work better [7]. Productivity is often expressed as a ratio of Gross Domestic Product to hours worked [12]. Increasing productivity has generally meant economies of scale, or investment in new and superior equipment, but increasingly it also means working smarter and enabling employees to make the best use of their time [13]. The latest advances in technology are enabling new modes of working, including smart-, tele-, remote-, or agile-working (terms often used interchangeably). This has become widespread in many industries. It might at first seem counterintuitive to imagine health sector employees working from home. Nonetheless, some professionals employed in health, including medical record transcribers or information technology (IT) professionals are already doing it on a pilot or permanent basis (one consideration is essential cybersecurity measures to protect the privacy of patient data). Health organizations offering this approach to working as a benefit for their employees include Mayo Clinic and Texas Health (both based in the United States (US)), which claim that it has helped them to increase employee satisfaction, and consequently to improve retention of existing employees, as well as to attract new talent [14]. It might be only a coincidence, but Texas Health was named best company to work for by the Dallas Business Journal, and one of the Top Best Companies by the Dallas Morning News in 2013 [15].

Employees, especially those of a younger age entering the workforce today, are using diverse devices and applications, beyond email, for efficient communication and collaboration. These include Slack or Workplace by Facebook. The former is already promising to “kill pagers” in the National Health Service (NHS), currently paying GBP 6,6 M per year for 130,000 pagers (making the organization the largest user of pagers in the world [16]). The latter launched recently with Facebook’s move into the corporate environment [17], and is already being used by some individual health organizations. Tan Tock Seng Hospital in Singapore, the first health organization in the world to trial Facebook Workplace, reports that it has simplified daily tasks such as for instance, requests for repairs (e.g. by sending a picture of a leaky air conditioner), publicly thanking employees, maintaining communication between course instructors and course participants, and conducting employee surveys. All of these are believed to be helping the hospital to achieve its mission of serving and treating people more effectively. Meanwhile, Australian Catholic University, after only four months of a pilot, has reported that 76% of employees felt that with Workplace their sense of participation improved, 63% declared that Workplace created more opportunities for collaboration, and 33% felt that the university’s channels of communication facilitated the sharing of project updates. Overall, a Sierra-Cedar HR technologies report [10] specific to the health sector revealed that between 2013 and 2014 the adoption by healthcare organization of social media tools was the most significant area of growth (24%) out of all technologies studied.

#### The introduction of continuous performance management processes

Performance management of healthcare employees can be vital to safeguarding quality of care and patient outcomes [18]. Moreover, performance management can often be used as a tool to engage employees and thus reduce turnover [18]. However, assessing the performance of a health workforce is a challenging task. Lack of standard procedures, the absence of measurable goals and inconsistent performance reviews are the most commonly mentioned causes for this [19]. Adoption of conventional HRIS helped some health organizations to launch the performance management process, and to address some of these challenges. For example, adoption of an electronic performance management HR system in Farer Park private tertiary healthcare institution in Singapore helped avoid misplacement or loss of paperwork by managers, reduce manual errors and lower total costs [20]. What is more, the next generation of performance management HRIS aim to go even as far as helping to align employee goals with overall organizational mission, to continuously monitor their achievements in real-time [21], and to evaluate performance not only at an individual level but also for a team (see “The maturation and growth of the “People Analytics” market” section for details). The latter is particularly important in healthcare, where not a single individual but a team of medical professionals is often responsible for patients’ treatment [22].

#### The rise of feedback, engagement, and analytics tools

Gallup’s recent study on the “State of the Global Workplace” [23] proclaimed that 85% of employees do not feel engaged at work. It was estimated that the associated loss of productivity might equate to as much as $7 trillion USD. The American Medical Association and the Mayo Clinic found that around 50% of healthcare providers demonstrated signs of burnout [24]. In health, a low level of employee engagement is often associated with high employee turnover or burnout, which can affect patient satisfaction and quality of care. For example, the engagement level of nurses was the number one predictor of patient mortality rates in 200 hospitals surveyed by Gallup [23]. On the other hand, health organizations getting employee engagement right have achieved notable results. At one hospital employee turnover declined to 15%, saving USD 1,7 M in reduced turnover cost and increased operating margin [24].

Overall, numerous tools are now available which enable health organizations to listen continuously to employees’ feedback, and to measure their engagement [25]. For convenience, these often come with practical user guidelines [26]. Finally, the recent acquisition of survey company Qualtrics by SAP opens up the possibility that these listening tools might be incorporated into HRIS or broader organizational Enterprise Resource Planning systems [27].

#### A reinvention of corporate learning

With the support of modern technologies, organizations are altering their training and development practices and methods to a remarkable extent. Some notable examples are discussed below.

Virtual and/or augmented reality (VR) technology is able to recreate complex situations and to make the learning experience entertaining. It is predicted to make its biggest impact on corporate and academic learning in healthcare [28]. Indeed, Microsoft, in collaboration with Case Western Reserve University, has already used the HoloLens holographic computer to develop a program that educates medical students in anatomy and illustrates the relationship between different body parts [29]. In a practical hospital setting, MPathic VR technology teaches medical students to improve their performance when empathetic communication skills are needed in delivering bad news to patients or relatives [30]. Meanwhile, Dr. Shafi Ahmed, Chief Medical Officer of Medical Realities, co-founded both this company and Virtual Medics in order to address the upcoming global shortages of surgeons by training large numbers of them simultaneously. One of Dr. Ahmed’s operations, recorded with Snapchat glasses, was viewed 2 million times and downloaded from YouTube by 100,000 people [30].

Pioneering “TV/Netflix-alike” training modules claim to offer a customized and personalized experience to employees. For health organizations, they promise to observe competencies on the job and to track clinical licenses and certifications in real-time, thus helping to increase compliance with the regulatory requirements of some government and accredited bodies [31].

Microlearning is also transforming how training can be delivered to busy health professionals, helping to address a lack of ongoing training for nurses - the primary reason why they tend to resign after a short time in the job [1]. Microlearning often consists of a series of short online videos that can be watched on smartphones at any convenient time. Mentis Neuro Health (US) claims that the adoption of this approach to teaching nurses how, for example, to transfer patients, helped them with: onboarding multiple employees in diverse geographical areas simultaneously; tracking health professionals’ training and education for compliance purposes; cutting expenses associated with employee travel and training venues; and eliminating dependencies on trainers, as well as differences in their teaching approaches [32].

#### Innovation in the recruitment market

Recruitment of healthcare professionals can be a lengthy process, taking up to 18–24 months [6, 33]. Considering the growing health workforce employment rate of 19% [34] and imminent staff shortages, healthcare organizations are revisiting their recruitment practices with the primary objective of finding and recruiting qualified personnel as quickly as possible. Indeed, even small changes to the recruitment process such as automating emails to candidates or the gathering of reference information have been shown to bring significant improvements. For example, the introduction of a recruitment checklist, giving details of what documentation to bring to interview for applicants of the Newcastle upon Tyne NHS Hospital Foundation Trust (UK) reduced recruitment time by more than half, saving 25,000 working days within 6 months of its introduction [6]. Meanwhile, by using the Healthcare Source Staff assessment solution, the Central Maine Medical Family (US) reported a reduction in turnover of employees in their first year from 22 to 15%, and of nurses from 20 to 11% [35].

Looking forward, technology giants such as Google (with Cloud Job Discovery) and LinkedIn (with TalentInsights) have recently launched a new generation of recruitment solutions. The former uses sophisticated classifications and relational models to match candidates’ preferences with relevant job announcements. The latter embraces the power of social media networking for recruitment. Moreover, recent strategic mergers including that between Workday HRIS vendor (used by circa 16% of health organizations [10]), and New York-based company Pymetrics, effected with a view to developing neuroscience- and AI-based assessment solutions suggest that traditional psychometric assessments used during recruitment are about to be transformed as they could be incorporated into traditional HRIS.

#### An explosion in the wellbeing market

There is a recognized relationship between employee wellbeing and organizational outcomes [36]. This is especially evident in healthcare, where wellbeing of the health workforce, as manifested in, for example, reduced absence days, can affect not only overall employee satisfaction but also have an influence on patients’ experiences and health outcomes [37]. Wearable devices are “taking the [health] industry by storm” in their aim to understand more about employee wellbeing parameters such as exertion, nutritional intake, sleep patterns, and stress levels [38]. It should be noted though that their use has been a matter of some public concern, and that employers need to find a balance in their efforts to achieve employee wellbeing and a potential lessening of insurance costs [39].

#### The maturation and growth of the “people analytics” market

In the era of big (and small) data, the status of employee data has become equal to that of customer data, since it is just as capable of providing descriptive, predictive or actionable insights that can improve organizational productivity. This practice of using employee data is called Workforce, HR, or more recently People Analytics [40]. Some associate it with the arrival of “geeks” and quantitative or qualitative evidence-based methods in the formulation of HR management protocols [41]. Instead of traditional HR reports, metrics, dashboards or scorecards reporting on staff turnover, employee absenteeism rates, or training budget expenditure per employee, now many organizations make HR-related decisions on who to hire, train or promote in a more strategic way. Other industries, with technology companies leading the way, have benefited especially from linking employee data with data coming from other organizational or external sources. These include sales, production, finance or social media data [40]. In health organizations, these additional data can be of clinical or administrative origin [9].

The problem of employee retention is not exclusive to the healthcare sector. It is common also to consulting, technological and pharmaceutical companies. Lead users from these sectors are actively applying the principles of People Analytics in addressing this challenge. The pilot results they have reported seem promising. Thus one of the Big Four consulting companies, E&Y, calculated that by improving turnover by 1% they can add USD 111 M to their bottom line [42]. Phizer also proactively predicts which employees are more likely to leave by drawing on scientific research that has established 13 signs that such employees manifest [43]. Similarly, in health, MultiCare Health System in the Pacific Northwest (US) testified that by using a commercial application scrutinizing information on existing employees and applicants they improved retention by 33%. Taking into consideration that replacement of every nurse can cost healthcare organizations’ from $37,700 to $58,400 [44], this initiative is definitely worthy of health leaders’ attention to ensure consistency of service to patients and to generate savings re-investable in other critical areas.

Traditional workforce management processes such as selection or performance assessment focalize on individual employees. However, teamwork is a critical feature of the modern workplace. But we know very little about how teams operate in health organizations. Indeed, consideration of how to identify teams (rather than departments) in health organizations or how to assess their performance is still scarce. This has prompted the rise of Organizational Network Analysis (ONA). Compared to traditional organizational charts illustrating only the hierarchical vertical roles of employees, used by organizations for over 70 years, ONA can help to understand the dynamics of complex interconnectivity in workflow exchanges at three levels: team (e.g. how individual nurse interacts within a team of nurses), organization (e.g. how nursing teams operate within health organizations), and enterprise (e.g. how individual health organizations operate within a larger health system). This innovation is already being used in health to analyze and improve the relationship of professionals within and across disciplines, work processes, and organizational structure in general [45]. The published evidence on the outcomes of these initiatives is still scarce, though training offerings on the use of ONA in health are expanding [46], and technological developments have even allowed the creation of a 3D ONA tool [47].

#### The spread of intelligent self-service tools

Tursunbayeva and colleagues [9] conducted a comprehensive literature review on HRIS in health. Analyzing around 7000 publications, they shortlisted 42 studies as of particular significance. Off these, only six mentioned development, implementation or use of HR self-service tools in health organizations. Nonetheless, these studies documented their usage all around the world, including Saudi Arabia, the Netherlands, Turkey, the USA, and the UK.

Meanwhile, Tower Watson’s study [11] reported that 59% of healthcare providers have HR Portals, although many of these need an update. They are also often lacking in usability and possibilities for personalization. Healthcare organizations which have redesigned their HR Portals have declared that in doing so they have been influenced by social media in incorporating features found on Google, LinkedIn, Facebook and the general environment of mobile phone operating systems.

Overall, the diffusion of self-service tools has empowered managers and employees to take responsibility for some HR practices previously performed by or with the support of HR managers. This has enabled a transformation in the role of HR divisions from being support functions to becoming business partners involved in strategic decision-making (see [48] for discussion).

#### Innovation within HR itself

Health organizations worldwide are moving to a new value-based operating model; shifting from fee-for-service to fee-for-outcome. This requires prioritization of efficiency, patient experience, and health outcomes. The role of HR in this business model is invaluable. However, to support this innovation HR functions need to complement their administrative HR activities with strategic practices recommended by the World Health Organization almost two decades ago [49]. These, however, need to be aligned with the broader strategy of the organization and backed up with HR technologies and actionable analytics. This transformation has taken place in many companies internationally over the last decades, and now the healthcare industry is catching up with this HR revolution. For example, as per Towers Watson’s 2014 HR Service Delivery and Technology Survey results [11], 52% of health care provider organizations have already shifted to a system-wide HR function, while 22% were designing new system-wide approaches for HR in 2014.

Strategic workforce planning has also emerged as a crucial HR practice in health organizations. It can provide insights regarding not only employee numbers, but also the ideal distribution of skills needed in a productive and effective team, or skills that organizations might need in the future. These can include complex statistical data analysis, digital or project management skills previously not much in demand in health organizations. Such skill inventorying is being actively promoted by IBM, which piloted this approach with circa 330,000 employees from diverse departments worldwide, who self-assessed their expertise against a pre-defined taxonomy [50].

Finally, with the growth of health workforce migration [51], and an increase in smart- and gig- workers in health, HR functions will also need to reconsider accordingly their HR and organizational development practices to ensure that they are inclusive and aimed at overseeing management of a more diverse and changeable talent pool.

### Debate

Different forms of administrative information systems including HRIS have been used in health organizations for the past five decades, although these often used to be de-prioritized in health organizations by comparison with clinical information systems. Rising concern regarding HRH shortages has increased interest in exploring the role of HR technologies in the management, development and drive for increased efficiency of HRH. However, many of the existing studies related to technology or its impacts on the future of work in healthcare have focused primarily on just a few specific technologies, especially AI [3, 52]. This is despite the promise that a wider and ever-expanding range of technologies, or combinations of technologies, can help to address various aspects of this crisis. A particularly interesting newcomer is Blockchain, which promises not only to facilitate HR data collection and storage but also to ensure its security and confidentiality. This is especially timely in the wake of the noteworthy hacks of 21.5 M records in the US’s Office of Personnel Management and 1.7 M records in the UK’s Ministry of Finance, as well a recent case in which a man was found to have been masquerading as a doctor for a decade, after faking his medical qualifications [53].

Many of the existing studies related to technology and its impacts on the future of work in healthcare also focus primarily on automation of work. Thus, for example, we know that the work of technicians, transcribers, secretaries, clinical coders and information technologists are likely to be computerized first [52]. Moreover, we can also deduce that it is likely that healthcare will have a mixture of automated, semi-automated, augmented and purely human roles. However, most of these studies often pay little or no attention to the fact that HR technologies are also likely to change how HRH are managed in health organizations.

The examples discussed in this paper illustrate how different HR technologies are helping or could help health organizations in improving their HR practices, and thus contribute to efforts to address the upcoming HRH crisis. However, while there is an evidence-base for the benefits and unintended consequences of operational and some strategic HRIS used in health organizations [9], there has been relatively little information and discussion regarding most of the disruptive HR technologies discussed above. The exceptions tend to derive from high-income contexts or private health organizations, making it difficult to assess whether and how the adoption or impact of these technologies really varies between geographies, cultures and other salient factors. However, it is promising that there are now some attempts to gather evidence regarding how these technologies or their applications can contribute to HRH productivity, especially from an international point of view. One significant example is the book “Human: Solving the Global Workforce Crisis in Healthcare,” written by the Global Chairman and Senior Partner for Healthcare, Government and Infrastructure at KPMG professional service company [13].

Most of the HR technology disruptions that have been discussed are still to find their place within health organizations’ routine HR practices. For example, none of the successful case studies featured on the website of LinkedIn Talent Solutions [54] comes from a health organization. This may be explained by the absence of an evidence-base that could be used to justify investments in HRIS, the focus of health organizations on adopting clinical IT or perhaps by some notable challenges HRIS developments and implementations face internationally, in a sector where a “mistake” can cost lives. The most notorious HRIS project ended up being described as “The largest admitted IT project failure in the Southern Hemisphere,” costing the Queensland (Australia) health system $1.25 billion AUD [55]. In the light of these considerations we need the collaboration of the academics and practitioners in developing new “programs of interdisciplinary research, encompassing economic evaluations, sociotechnical analyses, studies of information flows, and systematic assessments of the impacts of better workforce information on health care efficiency, quality, safety, and patient care, as well as new exploratory research to understand the value of information for driving analytics in support of sustainable and effective health systems” [9]. In the meantime, HRIS projects in healthcare should carefully pilot and evaluate incorporation of these technologies into complex health work practices in a way which take into consideration the readiness and maturity of individual organizations, [56] to including context-specific environmental, organizational and individual factors [48]. Moreover, the decision to adopt any particular type of HRIS should be considered with a clear eye on the specific organizational objective, whether this be an aspiration to collect accurate data on HRH (thus implying the adoption of core HRIS), or a desire either to introduce or to further transform strategic HR practices (demanding the adoption of performance management and recruitment HR modules, or even some disruptive HR technologies).

Finally, considering growing public awareness and concern about legal, compliance and ethical issues of data security and privacy, the success of projects related to HRIS or the use of their data may directly depend also on the responsible use of these innovations [40]. Some contexts have comprehensive regulations governing this, such as the European General Data Protection Directive. Others, waiting for their regulations to catch up, aim to fill this gap with ethical frameworks/policies. Diverse interdisciplinary scholars and practitioners are already actively working on addressing some of the ethical/legal issues related to adoption of HR and HRIS innovations [40]. However, practical ethical frameworks are still not available for all HR practices such as, for example, People Analytics, or if they exist they are not specifically tailored to health organizations. Overall, this is a big challenge, especially for developing countries where these technologies are often open-source and their adoption is funded by international sponsors.

## Conclusion

Although HRIS will not simply plug the need-capacity gaps in diverse health systems (e.g. NHS) [4], they can help by facilitating actionable analytics, above all by creating faster loops between data, discovery, and action. The balance among each of the technologies discussed above will vary according to their ability to connect, the quality of available data, the maturity of the algorithms underlying AI and the speed at which policies and regulations can adapt to accommodate these innovations in the safety-critical context of healthcare.

Each health organization will need to make an independent judgement regarding HR practices to be supported by HRIS, the type of HRIS to adopt, appropriate timing, and what stakeholders to involve in these projects. However, these decisions should be based not only on the availability of funding or conformity with lead-users, but also on the objectives specific to the organization in question, its preparedness to embrace these technologies [56], on context-specific environmental, organizational and individual factors, and on the evidence-base informing their development, implementation or use. Thus this evidence-base needs to be enlarged and enriched as soon as possible.

Finally, although these technologies promise many benefits, health organizations, health and HR professionals need to use them responsibly in a sector as complex and sensitive as healthcare. It will be vital to find a balance between the drive for innovation, efforts toward achieving productivity and efficiency, the imperative to respect all potential legal, ethical and compliance issues, and, last but not least, attending to HRH wellbeing and satisfaction.

## Abbreviations

AI: Artificial Intelligence
HR: Human resource
HRH: Human Resources for Health
HRIS: Human Resource Information Systems
IT: Information technology
NHS: National Health Service
ONA: Organizational Network Analysis
UK: United Kingdom
US: United States
VR: Virtual reality
